# Quorum sensing in group A Streptococcus

**DOI:** 10.3389/fcimb.2014.00127

**Published:** 2014-09-12

**Authors:** Juan Cristobal Jimenez, Michael J. Federle

**Affiliations:** ^1^Department of Microbiology and Immunology, College of Medicine, University of Illinois at ChicagoChicago, IL, USA; ^2^Department of Medicinal Chemistry and Pharmacognosy, Center for Pharmaceutical Biotechnology, College of Pharmacy, University of Illinois at ChicagoChicago, IL, USA

**Keywords:** quorum sensing, pheromones, cell-cell signaling, *Streptococcus pyogenes*, Rgg, Sil, lantibiotics, AI-2

## Abstract

Quorum sensing (QS) is a widespread phenomenon in the microbial world that has important implications in the coordination of population-wide responses in several bacterial pathogens. In Group A Streptococcus (GAS), many questions surrounding QS systems remain to be solved pertaining to their function and their contribution to the GAS lifestyle in the host. The QS systems of GAS described to date can be categorized into four groups: regulator gene of glucosyltransferase (Rgg), Sil, lantibiotic systems, and LuxS/AI-2. The Rgg family of proteins, a conserved group of transcription factors that modify their activity in response to signaling peptides, has been shown to regulate genes involved in virulence, biofilm formation and competence. The *sil* locus, whose expression is regulated by the activity of signaling peptides and a putative two-component system (TCS), has been implicated on regulating genes involved with invasive disease in GAS isolates. Lantibiotic regulatory systems are involved in the production of bacteriocins and their autoregulation, and some of these genes have been shown to target both bacterial organisms as well as processes of survival inside the infected host. Finally AI-2 (dihydroxy pentanedione, DPD), synthesized by the LuxS enzyme in several bacteria including GAS, has been proposed to be a universal bacterial communication molecule. In this review we discuss the mechanisms of these four systems, the putative functions of their targets, and pose critical questions for future studies.

## Bacterial communication in gram-positive bacteria

For a long time, bacteria were thought of as organisms carrying out self-sufficient and independent, unicellular lifestyles. During the last 40 years, several studies have demonstrated how, in fact, bacteria interact and establish complex social behaviors with their siblings and with other bacteria in their community to develop beneficial actions for the population, by means of conserved chemical languages. Quorum Sensing (QS) is the communication process in which bacteria produce, secrete and detect chemical signals with the purpose of triggering specific phenotypical responses. QS regulates genes involved in population-wide decisions and behaviors that are beneficial when performed as a synchronous group rather than at the individual level and which include bioluminiscence, sporulation, competence, antibiotic production, biofilm formation, and secretion of virulence factors (Reviewed by Atkinson and Williams, 2009; Ng and Bassler, 2009; Rutherford and Bassler, 2012).

**Figure 1:**
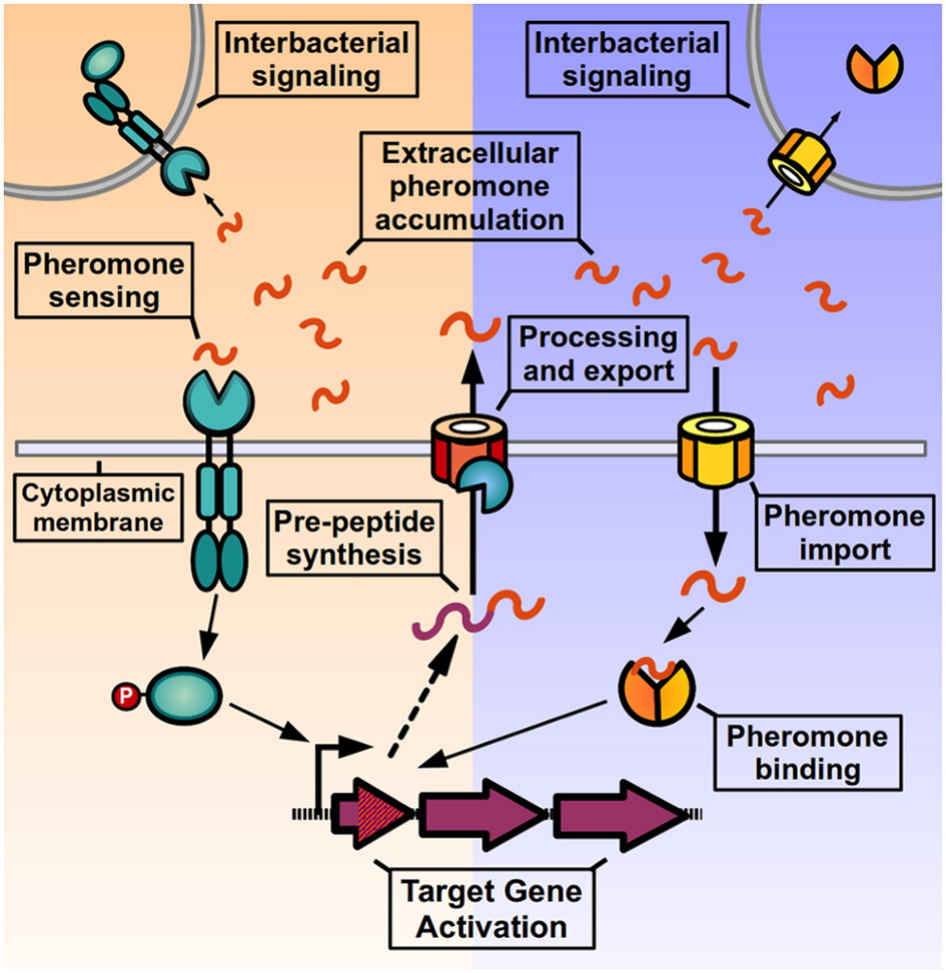
**Quorum sensing signaling in Gram positive bacteria**. After being translated by the ribosome, pre-peptides are processed, and exported from the cell to generate an active signaling pheromone. Pheromones accumulate in the extracellular medium where they can be detected by the producer cell and neighboring bacteria. Pheromone detection can either occur through two-component systems in the bacterial membrane (left side) or by direct binding by transcription factors after peptide import (right side). After pheromone detection, the activated response regulator or the pheromone-bound transcription factor induce changes in target gene expression, and genes encoding for pheromone pre-peptides are up-regulated, increasing pheromone production and generating an autoinduction process.

QS signaling in Gram-positive bacteria (Figure 1) operates through the activity of post-translationally modified oligopeptides, named autoinducing peptides or pheromones, which can range from 5 to 34 amino acids in length and can adopt either linear or cyclical conformations (Håvarstein et al., 1995; Ji et al., 1995; Kuipers et al., 1995; Solomon et al., 1996; Otto et al., 1998; Mayville et al., 1999; Sturme et al., 2005). These pheromones are initially synthesized as inactive pro-peptides in the ribosome, and then exported from the cell by either the general secretion system (Sec) or by dedicated ABC transporters (Hui and Morrison, 1991; Zhang et al., 2002; Stephenson et al., 2003). During the export event, pro-peptides undergo proteolytic processing (and in some cases additional covalent modification) to generate the active pheromone, and a variety of enzymes have been involved in these maturation processes (Magnuson et al., 1994; Otto et al., 1998; An et al., 1999; Mayville et al., 1999; Zhang et al., 2002; Lanigan-Gerdes et al., 2007; Thoendel and Horswill, 2009). When the pheromones surpass threshold concentrations in the extracellular medium they are efficiently detected by transmembrane receptors of the two-component system (TCS) signal transduction family, leading to differential phosphorylation of a response regulator and consequent change in target gene expression. Alternatively, pheromones can be imported into the cytoplasm via peptide transporter complexes, most commonly the Opp/Ami oligopeptide permease, a promiscuous transporter of peptides involved in the import of nutritional peptides, peptidoglycan recycling components as well as pheromone peptides for other QS systems (Leonard et al., 1996; Lazazzera et al., 1997; Slamti and Lereclus, 2002; Fontaine et al., 2010; Mashburn-Warren et al., 2010; Chang et al., 2011). Once inside the cell, peptide pheromones bind and directly modulate the activity of transcriptional regulators inside the cell. As a result of signaling, target genes change their expression pattern and genes encoding for the pheromone pre-peptides are upregulated, increasing the production of mature pheromone and generating a positive-feedback loop (or autoinduction process), that helps strengthen the QS signaling at the individual level and increases pheromone levels in the environment to activate signaling at the population level.

Among the earliest reports describing cell-to-cell communication in bacteria arose from studies of genetic exchange in *Streptococcus pneumoniae*, where it was shown that a “hormone-like cell product” secreted by the bacteria into the culture medium activated their ability to import extracellular DNA and undergo genetic transformation in a process termed competence (Tomasz, 1965). Several years later, the signaling molecule was shown to be a processed peptide named CSP (competence-stimulating peptide), that formed part of a quorum-sensing circuit (Håvarstein et al., 1995). Since then, several pheromones and their associated QS systems have been discovered and characterized in species of the *Streptococcus* genus (Reviewed by Cook and Federle, 2014). The existence of QS systems in *Streptococcus pyogenes*, a human-restricted pathogen capable of causing a broad spectrum of diseases, was unknown until recently. Also known as Group A *Streptococcus* (GAS), *S. pyogenes* is a polyauxotrophic organism that resides primarily in the oropharynx and the skin of its human host and generates illnesses that range from mild, like pharyngitis and impetigo, to severe and life threatening, like necrotizing fasciitis and toxic shock syndrome. GAS presence in the host does not necessarily correlate with a disease state, as it can be isolated from several body sites in asymptomatic individuals and is carried asymptomatically in approximately 20% of school-aged children (Kaplan and Huwe, 1980; Mead and Winn, 2000; Shaikh et al., 2010; Roberts et al., 2012). GAS is an organism that develops its complete life cyle inside the human host, passing from non-virulent asymptomatic colonization to symptomatic infection, to transmission and dissemination (Wollein Waldetoft et al., 2014). Because of this, it is of great interest to understand the ways by which GAS populations regulate their collective behaviors, and how these behaviors may be associated with adaptation to the host environment or with the switch between pathogenic and non-pathogenic states. In this review we examine the mechanism and function of four families of QS systems present in GAS: regulator gene of glucosyltransferase (Rgg), Sil, lantibiotic regulatory systems, and AI-2.

## Rgg transcriptional regulators

In Gram-positive bacteria, two families of conserved transcription factors have been reported to interact with imported peptide pheromones: RNPP and Rgg. The RNPP family, named for its prototypical members Rap, NprR, PlcR, and PrgX, is characterized by the presence of tetratricopeptide repeat domains (TPRs) in their C-terminal domains, defined by motifs that are involved in protein-protein interactions and are involved in peptidic pheromone binding (Blatch and Lässle, 1999; Core and Perego, 2003). Some members of the RNPP family also possess Helix-Turn-Helix (HTH) motifs for DNA binding and direct regulation of gene expression (Declerck et al., 2007; Rocha-Estrada et al., 2010). RNPP Representatives are found in *Bacillus* and *Enterococcus* species and have been shown to regulate processes of sporulation, conjugation, biofilm formation and pathogenic responses (Rocha-Estrada et al., 2010). The second conserved group of peptide-binding transcription factors is the Rgg family. Members of this family possess characteristic HTH motifs in their N-terminal domains and a C-terminal region rich in alpha-helical structures. Rgg members possess a low level of sequence similarity with members of the RNPP family and their C-terminal domains, presumed to bind peptide pheromones, have not been characterized structurally so far, and no crystalographic data of Rgg members is available yet (Fleuchot et al., 2011). The Rgg family members are widespread in the low-G+C Gram-positive bacteria and only absent in the *Clostridiaceae* (Fleuchot et al., 2011). Rgg proteins have been shown to behave like activators or repressors of DNA expression, while some can exhibit simultaneously both regulatory functions (Rawlinson et al., 2002; Samen et al., 2006; Anbalagan et al., 2011). The first Rgg family member was identified in the oral bacterium *Streptococcus gordonii*, in which extracellular glucosyltransferase activity required for tooth surface colonization was shown to be promoted by the activity of the Rgg protein (Sulavik et al., 1992). Since then, several other Rgg members have been characterized in Streptococcal species including *S. oralis* (Fujiwara et al., 2000), *S. thermophilus* (Fernandez et al., 2006; Ibrahim et al., 2007a; Fontaine et al., 2010), *S. salivarius* (Fontaine et al., 2010), *S. pneumoniae* (Bortoni et al., 2009), *S. mutans* (Qi et al., 1999), *S. agalactiae* (Samen et al., 2006), and *S. suis* (Zheng et al., 2011). Some species may even harbor multiple *rgg*-like genes in their genomes, like the case of *Streptococcus thermophilus*, predicted to encode in its genome seven different Rgg paralogs (Ibrahim et al., 2007b). After the discovery that the deletion of a small pre-peptide gene inhibited the regulatory activity of an Rgg protein in *S. thermophilus*, it was recognized that activity of Rgg regulators was modulated by short peptides, constituting putative QS circuits (Ibrahim et al., 2007a). Commonly, Rgg genes are located next to a short open reading frame that encodes the propeptide of their cognate pheromone, short genes which are usually overlooked in genome annotation processes but have been predicted by *in silico* analysis (Ibrahim et al., 2007b; Fleuchot et al., 2011). Rgg pheromones have been classified in two groups to date, short hydrophobic peptides (SHPs) and peptides involved in competence pathways, termed XIPs (Table 1) (Mashburn-Warren et al., 2010; Fleuchot et al., 2011). Since some of the Rgg/pre-peptide loci show high conservation among different streptococci, it has also been shown that interspecies cross-talk can occur via SHP-pheromones (Cook et al., 2013; Fleuchot et al., 2013). In GAS, four Rgg paralogs can be identified based on sequence homology: RopB (Rgg), Rgg2, Rgg3, and ComR (Rgg4) (Chang et al., 2011; Federle, 2012).

**Table 1:** **GAS Pheromones**.

**Pre-peptide (pheromone)**	**Sequence**	**References**
SHP2 **(SHP2-C8)**	MKKISKFLPILILAM**DIIIIVGG**	Chang et al., 2011
SHP3 **(SHP3-C8)**	MKKVNKALLFTLIM**DILIIVGG**	Chang et al., 2011
ComS M1 **(XIP)**	MLKKYKYYFIFAALLSFKVVQEL**SAVDWWRL**	Mashburn-Warren et al., 2012
ComS M3 **(XIP)**	MLKKVKPFLLLAAVVAFKVARVMH**EFDWWNLG**	Mashburn-Warren et al., 2012
SilCR	MNNKKTKNNFSTLSESELLKVI*GG***DIFKLVIDHISMKARKK**	Hidalgo-Grass et al., 2004
Salivaracin A **(SalA1)**	MSFMKNSKDILTNAIEEVSEKELMEVA*GG***KKGSGWFATITDDCPNSVFVCC**	Upton et al., 2001
Streptin **(Streptin 1a)**	MNNTIKDFDLDLKTNKKDTATPY**VGSRYLC*T*PGSCWKLVCF*TTT*VK**	Wescombe and Tagg, 2003
Streptococcin A-FF22	MEKNNEVINSIQEVSLEELDQII*GA***GKNGVFK*T*I*S*HECHLN*T*WAFLA*T*CCS**	Jack et al., 1994b

*Amino-acid sequences of pheromone pre-peptides and predicted cleaved pheromones (in bold). Double-glycine and glycine-alanine cleavage motifs are italicized and underlined. Pheromone residues that undergo dehydration reactions are bold and italicized, and residues believed to form thioether bridges are bold and underlined*.

### RopB

**Figure 2:**
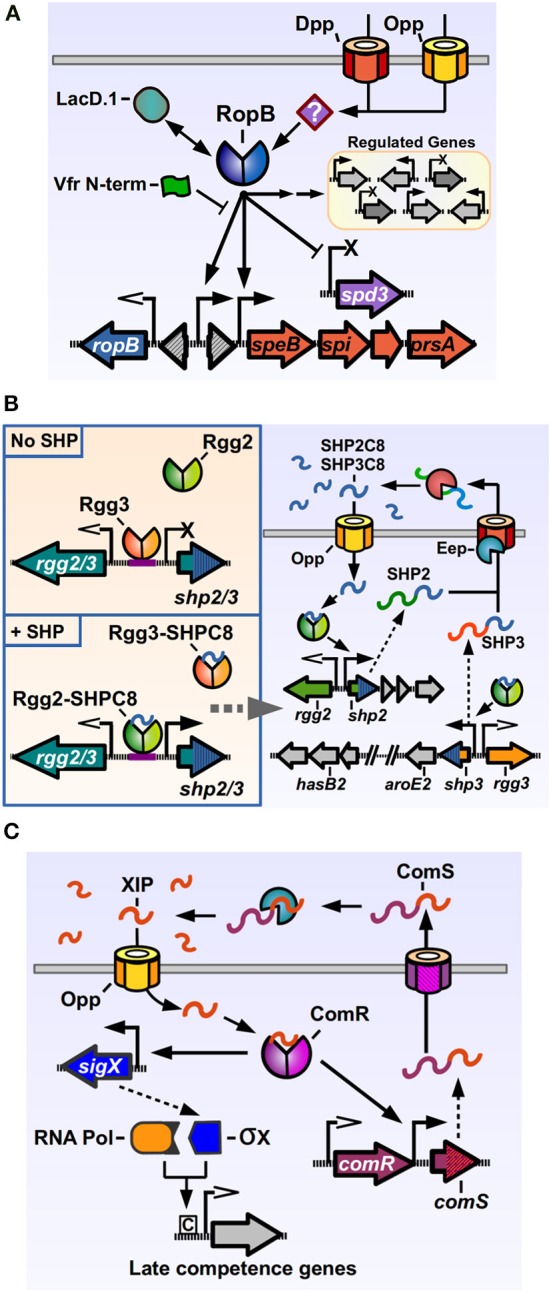
**Rgg regulators of *Streptococcus pyogenes*. (A)** RopB directly activates the expression of *speB* and its associated downstream genes, while directly repressing the prophage encoded *spd3* DNase. RopB also affects, by direct and indirect manners, the expression of a varying group of genes in different isolates. Other factors can modulate RopB activity, like LacD.1, the N-terminal peptide of the Vfr protein and unknown factors imported by the Opp and Dpp permeases. **(B)** The Rgg2/3 system. Left panel: in the absence of SHPs, Rgg3 is bound to the promoter region of the pheromone genes, inhibiting their expression. Addition of exogenous SHP pheromones that bind Rgg3, cause its release from DNA, while allowing Rgg2 to bind the same promoter region and promote expression of pheromone genes. Right panel: activation of the Rgg2/3 system triggers expression of *shp2*, *shp3* and their downstream genes. Translated SHP2 and SHP3 pro-peptides are secreted through an unknown exporter and processed by the activity of Eep and additional extracellular enzymes. The active SHP2-C8 and SHP3-C8 pheromones are imported via Opp to complete the autoinduction loop. **(C)** Regulation of competence genes by ComR. ComS pro-peptide is produced, secreted and processed to generate the XIP peptide. After being imported through Opp, XIP can bind ComR, which binds to the promoters of *comS* and *sigX* to activate their expression. The alternative sigma factor σX together with RNA polymerase bind to Com box sites in target genes and activate the expression of competence related genes. Promoters with open arrows are Rgg-independent. Promoters with filled arrows are activated by Rgg-proteins. Promoters with X symbol are repressed by Rgg-proteins.

The most studied Rgg member of GAS is the RopB protein. One of the important GAS “stand-alone” regulators, transcription factors whose cognate sensor partners are unknown (Kreikemeyer et al., 2003), RopB, a predicted dimeric protein, has been shown to both positively and negatively modulate the expression of several genes (Figure 2A) (Chaussee et al., 2002, 2003, 2004, 2008; Dmitriev et al., 2006, 2008; Hollands et al., 2008; Carroll et al., 2011). RopB was initially discovered in a mutant screen for regulators of the potent cysteine protease SpeB. The streptococcal pyrogenic exotoxin B, SpeB, targets a wide range of proteins from the host, including components of the extracellular matrix and modulators of the immune response, as well as GAS proteins present in its envelope or secreted into the extracellular medium (reviewed by Chiang-Ni and Wu, 2008; Nelson et al., 2011). Hence, SpeB plays an important role in modifying the host response and in reshaping the bacterial surface, a fact that is reflected in the number of factors involved in its regulation (Carroll and Musser, 2011). RopB is essential for *speB* transcription, and regulates its expression by directly binding the distal P1 promoter in the intergenic region between the *ropB* and *speB* genes. As with other genes regulated by activation factors, the *speB* P1 promoter lacks a canonical −35 promoter sequence, which may result in poor binding by RNA polymerase and low expression in absence of the transcriptional activator. Located upstream of the −35 region of P1 are a two inverted repeat sequences, common recruitment motifs for dimeric DNA-binding proteins, which RopB may interact with in order to bind and recruit polymerase and activate *speB* expression, consistent with the activity of a type II activator (Neely et al., 2003; Browning and Busby, 2004). How RopB represses the expression of target genes is less understood, but it has been shown to be able to bind two different promoters in the upstream region of the repressed, prophage encoded *spd-3* gene, which possess −35 sequences closer to the consensus, suggesting that in this case RopB binding blocks the promoter region, inhibiting polymerase recruitment (Anbalagan and Chaussee, 2013).

Even though it is a member of the Rgg family, RopB has not been widely characterized as a pheromone binding protein or as a QS component, as it lacks characteristics reported in other Rgg-pheromone systems. No obvious pheromone encoding gene has been described near the *ropB* gene. Additionally, several studies on SpeB production and mutagenesis screens performed to understand its regulators in GAS have revealed a variety of factors modulating RopB activity, but so far no pre-peptide encoding ORF. Nonetheless, several results have shown that RopB activity is affected by interaction with peptides and proteins: The regulation of *speB* expression by RopB has been shown to be dependent on cell cycle, with the highest degree of expression beginning in the stationary phase, suggesting a requirement for high cell density conditions for RopB-mediated gene activation. If *ropB* is expressed from a non-native promoter at high levels during early exponential phase, *speB* transcription is still not activated, implying that additional factors are required for RopB regulation (Neely et al., 2003). Mutations in Opp or its paralog Dpp (dipeptide permease) generate a drastic decrease in *speB* expression, suggesting that peptide import into the cell is also required for *speB* regulation (Podbielski et al., 1996; Podbielski and Leonard, 1998; Wang et al., 2005). Other results have shown the role of proteins that directly interact with RopB to affect its regulatory function: LacD.1 is an aldolase enzyme that has been proposed to link metabolic status of GAS with virulence gene regulation, as it is able to bind intermediates of the glycolytic pathway that are present in high concentrations during exponential growth and consequently influence gene expression (Loughman and Caparon, 2006b). LacD.1 and RopB can be co-precipitated, and in absence of LacD.1 a constitutive *ropB* allele is able to partly uncouple SpeB activity from cell cycle regulation. Other reports, based on the observation of differential gene regulation by RopB during stages of bacterial growth, have suggested that LacD.1 binds to RopB during exponential phase, changing its DNA binding preferences and affecting the repertoire of genes regulated (Anbalagan et al., 2012). Another protein involved in repressing RopB-dependent SpeB activation is Vfr (virulence factor related) (Ma et al., 2009). The *vfr* gene was identified by transposon mutagenesis, as a mutant with increased protease activity, and SpeB activity is inversely correlated with *vfr* expression; *vfr* expression is maximal in early logarithmic stages and decreased toward stationary phase. Additional work by Shelburne et al. (2011) showed that the N-terminal region of the Vfr protein, which forms part of a putative cleavable secretion signal, was able to bind RopB and was sufficient to exert the inhibiting activity. However, a factor accounting for a necessary activating signal of SpeB regulation by RopB has yet to be identified.

Studies on the scope of RopB regulation have revealed a wide range of variation in the number of controlled genes depending on the studied GAS isolate (Table 2). In the strain NZ131, which has been characterized in several studies of RopB regulated phenotypes, the deletion of *ropB* generates a pleiotropic effect, affecting the expression of 293 genes during exponential phase, and changing the expression of 567 genes during post-exponential phase. These include genes involved in metabolism, stress response, prophage excision, and virulence (Dmitriev et al., 2006). These gene changes reflect on several phenotypes shown for NZ131 Δ*ropB* mutants, including increased secretion of DNases MF1 and Spd3/MF3, decreased secretion of ClpB chaperone, lysozyme and autolisin (Watson et al., 2001); increased production of M protein and the virulence factors C5a peptidase, Streptolysin S, and Streptolysin O (Chaussee et al., 2002); increased catabolism of arginine and serine (Chaussee et al., 2003); inability to utilize alternative carbon sources (Dmitriev et al., 2006) and increased resistance to H_2_O_2_ (Pulliainen et al., 2008). It has been thought that this pleiotropic effect is based upon the ability of RopB to influence the expression of other regulatory factors, as it has been shown to increase the expression of *covRS* TCTS, while repressing the expression of *mga* and *sagA* stand-alone regulators (Chaussee et al., 2002). However, when ChIP-on-chip analyses were performed, revealing 125 RopB binding sites in the NZ131 genome, it was noticed that none of the promoters of these regulators were bound directly by RopB (Anbalagan et al., 2011). These results highlight the fact that pleiotropic effects are not a consequence of direct RopB regulation, and that additional factors, including regulation of uncharacterized putative regulators by RopB (*cpsY*, *Spy49_1761*, *Spy49_1113*) (Anbalagan et al., 2011), or the induction of prophage genes and concomitant phage excision, may play a role in the widespread gene induction events after *ropB* deletion. When comparing different strains in which the RopB regulon has been analyzed, *speB* and its related co-transcribed neighboring genes (*spi*, a putative SpeB inhibitor; *prsA* peptidyl-prolyl isomerase involved in SpeB processing and SPy2040, a hypothetical gene) appear as the only genetic region consistently regulated (Kagawa et al., 2005; Ma et al., 2006; Dmitriev et al., 2008; Carroll et al., 2011).

**Table 2:** **RopB regulated genes**.

**Strain (M type)**	**Genes that change (% genome)**	**Genes decreasing expression**	**Genes increasing expression**	**References**
**NZ131 (M49)^a^**	567 (31)	340	227	Dmitriev et al., 2006
**CS101 (M49)^a^**	13	3	10	Dmitriev et al., 2008
**SF370 (M1)^a^**	45 (2.5)	17	28	Dmitriev et al., 2008
**MGAS5005 (M1)^a^**	3 (0.15)	2	1	Dmitriev et al., 2008
**GAS5448 (M1T1)^b^**	99	47	52	Hollands et al., 2008
**MGAS10870 (M3)^a^**	479 (25)	27	452	Carroll et al., 2011
**MGAS9937 (M3)^a^**	159 (8)	13	146	Carroll et al., 2011

*Gene expression changes in ΔropB strains compared with wild type strains as shown by microarray analyses*.

a *Post-exponential/early stationary phase*.

b *In vivo*.

Deletions in the *ropB* gene, or mutations that generate non-functional RopB proteins confer changes in GAS virulence, but the exact role of RopB regulation and its contribution over the infective process remains perplexing. This, due to contradictory results in the literature, is a consequence of the wide variation seen in the RopB regulon from different GAS isolates: Some studies show virulence to be reduced (Hollands et al., 2008; Carroll et al., 2011), while others show virulence is increased in RopB mutants (Pulliainen et al., 2008; Ikebe et al., 2010). Interestingly, several allelic variants of the *ropB* gene exist and a significant percent of clinical strains carry different SNPs in *ropB*: a study of 1178 M3 clinical isolates revealed that 28% of them carried polymorphic variants from the wild type gene (Olsen et al., 2012). The majority of *ropB* SNPs have been shown to inhibit its ability to promote SpeB production, while some SNPs have been shown to change the specificity of RopB regulation, affecting the binding of select promoters (Kappeler et al., 2009). It has been proposed that GAS genotypic and phenotypic heterogeneity contributes to distinct disease manifestations, and that mutations in certain genetic regulators are involved in the transition from non-invasive to an invasive phenotype (Sumby et al., 2006). The distribution of RopB variants is indeed skewed among GAS isolates, and invasive-disease isolates carry these SNPs in a higher proportion when compared with non-invasive or pharyngeal isolates, and *ropB* together with *covRS* were found to be the genes most commonly mutated in M3 invasive isolates (Beres et al., 2010; Ikebe et al., 2010; Carroll et al., 2011). Overall, this Rgg member has remained most closely tied to virulent phenotypes with a complex regulatory pattern, and the possibility for it to bind and respond to signaling pheromones remains only a theoretical possibility.

### Rgg2 and rgg3

The *rgg2* and *rgg3* genes were identified after a search for additional Rgg orthologs in the GAS genome (Chang et al., 2011). Rgg2 and Rgg3 proteins share a high degree of similarity (55% identical, 76% similar), and are encoded divergently from the genes for the pro-peptide pheromones which are termed SHP2 and SHP3 respectively (Figure 2B). Both SHP2 and SHP3 pro-peptides are also highly similar (58% identical, 62% similar), 23 and 22 amino acids long, respectively, and contain almost identical C-terminal regions (Table 1). Basal expression of SHP2/3 pheromones is repressed under normal laboratory culturing conditions, suggesting that specific environmental signals are required to activate the QS system endogenously. Addition of synthetic SHP2 or SHP3 full length pro-peptides are able to generate a small increase in expression from the P*shp2* and P*shp3* promoters, consistent with an autoinducing signaling system, and addition of the last eight C-terminal amino acids of SHP2 or SHP3 (termed SHP2-C8 and SHP3-C8) generate a strong inducing activity (Chang et al., 2011). Further studies have shown that multiple variants of SHP2 and SHP3 pheromones can be found in GAS culture supernatants, corresponding to the C7, C8, C9, and C10 regions of the pheromone pro-peptides, and that the SHP-C8 variants of the pheromones are the most abundant and most biologically active forms (Aggarwal et al., 2014). These results also show that both SHP2 and SHP3 signaling peptides have the same function of activating the Rgg2/3 circuit, in comparison with other dual pheromone systems were one peptide acts as an activator and the other as a repressor of the QS circuit (Nakayama et al., 1994). If a strain that lacks both *shp2* and *shp3* genes is used, the induction of the system by synthetic pheromones is only transient, suggesting that the autoinduction process is required to generate the full extent of the response (LaSarre et al., 2013). Even though they seem redundant in function, both *shp2* and *shp3* genes are required for efficient signaling and autoinduction processes, as mutations in either of them affects the timing and breadth of the signaling response (LaSarre et al., 2013). Interestingly, even though other streptococci carry homologs of the *rgg2-shp2* or the *rgg3-shp3* loci (discussed below), to date GAS is the only sequenced streptococcal species that carries both *rgg2-shp2* and the *rgg3-shp3* loci, suggesting that this bacterium has evolved to use both regulators and their two cognate pheromones. As reported for other Rgg systems, an intact Opp system is required in order to import the pheromones and trigger activation of the system. Experiments also revealed that a deletion of the *rgg3* gene derepresses expression of the *shp2* and *shp3* genes, while a double mutant Δ*rgg2* Δ*rgg3* lacks nearly all expression of pheromones. A single deletion of *rgg2* also renders GAS grossly unresponsive to synthetic SHPC8 pheromones, illustrating how Rgg3 acts as a repressor of gene expression, while Rgg2 activates gene expression in the absence of the repressive effect of Rgg3 (Figure 2B). Deletion of the *eep* gene, encoding for a metalloprotease involved in pheromone processing in other Gram-positive QS systems, reduces the effectiveness of the autoinduction process, suggesting a role in the processing of SHP2/3 pre-peptides into their mature form. This effect can however be ameliorated if SHP3 is overexpressed, suggesting that additional proteins are involved in SHP2/3 processing (Chang et al., 2011).

Both Rgg2 and Rgg3 have been shown to bind directly to the promoter regions of *shp2* and *shp3* (Chang et al., 2011; LaSarre et al., 2012). Interestingly, when the precise DNA binding sites of both regulators were mapped by DNase I footprinting, Rgg2, and Rgg3 where shown to bind to the same conserved sequence in the P*shp2* and P*shp3* promoters, hence only one Rgg protein is able to bind per promoter at any given time, indicating that Rgg3 exerts it repressing activity by binding the shared DNA site and generating steric interference that inhibits Rgg2 binding (LaSarre et al., 2012). This is consistent with the fact that the HTH domains of Rgg2 and Rgg3 are distinctively analogous, sharing 71% identity and 94% similarity. A higher degree of variability is seen in the C-terminal regions of the Rgg2 and Rgg3 proteins, which are predicted to be involved in protein oligomerization and interaction with RNA polymerase, and this variability may determine the fundamental differences in the activities of these two regulators (LaSarre et al., 2012). Similar to the RopB interaction with the *speB* promoter, Rgg2 and Rgg3 bind the P*shp2* and P*shp3* promoters in their −35 regions, which have poor resemblance with consensus sequences, suggestive of a class II activator dependent promoter (LaSarre et al., 2012). Rgg2 and Rgg3 also directly bind mature SHP2 and SHP3 pheromones, exhibiting a higher affinity for the SHPC8 pheromone variants, while not binding full-length pro-peptides (Aggarwal et al., 2014). Using EMSA assays, it was revealed that either Rgg can bind the target DNA sequences in the absence of pheromones. However, when pheromones are added in increasing concentrations, Rgg3 is released from the DNA while Rgg2 remains unaffected. When the experiment is set up with both regulators to mimic the DNA-binding competition event *in vitro*, addition of synthetic pheromones affects the binding ratio, which becomes skewed in favor of Rgg2-DNA interactions over Rgg3-DNA interactions (LaSarre et al., 2012).

The function of the Rgg2/3 circuit and its contribution to the GAS lifestyle has not been completely elucidated. Apart from the *shp2* and *shp3* genes, other major regulatory targets of the Rgg2/3 pathway are the genes located downstream of the pheromone genes (Figure 2B) (Chang et al., 2011). Downstream of *shp3*, a putative biosyntethic operon composed of nine genes is encoded, which harbors genes with diverse enzymatic functions, like shikimate dehydrogenase, sugar isomerase, glycosyltransferase, and oxidoreductase. Components from this operon have been identified in mutagenesis screens for genes affecting mucoid colony morphology, virulence in zebra fish, and have also been involved in capsule formation in GAS (Biswas and Scott, 2003; Kizy and Neely, 2009; Cole et al., 2012). A putative locus encoding homologs of *rgg3-shp3* and its downstream associated genes are also present in *Streptococcus pseudoporcinus* and *Bacillus thuringensis* species. In the latter, an operon that encodes genes with similar enzymatic functions has been implicated in the production of thuringiensin, a wide-range insecticidal toxin predicted to inhibit RNA polymerase (Liu et al., 2010). Downstream of *shp2*, Rgg2/3 also controls a highly conserved region in GAS, which encodes a series of hypothetical genes of unknown function. This region however is required for a SHP-dependent increase in biofilm formation seen in NZ131 when the Rgg2/3 system is activated (Jimenez, unpublished results). We are currently working to elucidate the mechanism by which this this system contributes to biofilm formation. Homologous loci encoding *rgg2-shp2* with its downstream-associated genes are conserved in species of the pyogenic group streptococci, including *S. canis*, *S. agalactiae, S. dysgalactiae* subspecies *dysgalactiae* and *equisimilis* and *S. iniae*. The *S. agalactiae* Rgg2 homolog, called RovS, has been involved in regulating expression of genes required for epithelial cell attachment and hemolysis activity (Samen et al., 2006), and we have recently shown that this transcription factor is also modulated by a SHP pheromone and that both GAS and *S. agalactiae* (also called Group B Streptococcus, GBS) can undergo interspecies QS signaling when co-cultured (Cook et al., 2013). Since GAS and GBS can be isolated from the same sites in the human host, it is intriguing to wonder if these common signaling pathways constitute a way for these two organisms to coordinate intraspecies behaviors. Alternatively, and since other pyogenic group species that carry *rgg2-shp2* homologs reside in other mammalian species, this system may serve as conserved and efficient way to control gene expression that has evolved to control specific traits in each organism.

### ComR

ComR is another Rgg member present in GAS, involved in natural genetic transformation processes in other streptococci through the regulation of early genes in the competence cascade (Figure 2C). Competence, the temporal physiological state of DNA receptivity, relies in the coordinated and sequential expression of a series of genes, encoding proteins responsible for pheromone signaling, DNA binding, uptake, processing and recombination into the chromosome (reviewed by Johnsborg et al., 2007). In the streptococci, the master regulator of competence is SigX/ComX, an alternative sigma factor of RNA polymerase that recognizes a conserved DNA sequence termed cinbox or combox and controls the expression of the competence “late genes” required for DNA binding, uptake and recombination (Lee and Morrison, 1999; Peterson et al., 2000; Luo and Morrison, 2003). All the genes induced before *sigX* in the competence cascade and that are involved in the signaling processes that lead to *sigX* expression are termed “early genes.” In *S. pneumoniae*, the expression of *sigX* is under the control of a QS system, composed of the ComDE TCS which senses and responds to the competence inducing pheromone CSP. GAS and other streptococci of the pyogenic, bovis, and salivarius groups lack ComDE but have an alternative circuit, based on ComR activity, to trigger *sigX* expression and concomitant upregulation of late genes in the bacterial population. ComR was first discovered in *Streptococcus thermophilus*, a species from the salivarius group that is able to express competence genes and undergo natural transformation in an Opp-dependent manner when grown in peptide-free chemically defined medium (CDM), suggesting that import of self-produced peptides was essential for transformation (Gardan et al., 2009). To identify the regulators of *sigX* in *S. thermophilus*, the transcriptome of a mutant unable to express any competence genes (Δ*opp* mutant) was compared with a strain only able to express early competence genes (Δ*sigX* mutant). One of the identified components was a previously identified *rgg*-pheromone gene pair which were then shown to be required for *sigX* expression and natural transformation (Ibrahim et al., 2007a; Gardan et al., 2009). The regulator was named ComR for competence regulator and the pro-peptide gene was called *comS* for competence signal (Fontaine et al., 2010). The *sigX* promoter, bound by ComR, lacks a canonical −35 sequence and has an inverted repeat element in this region, suggestive with a Class II activator dependent promoter, similar to the P*shp2/3* promoters activated by Rgg2 and the P1 *speB* promoter activated by RopB (Mashburn-Warren et al., 2010). *In silico* screening in streptococcal genomes revealed the presence of *comRS* orthologs in the genomes of pyogenic, bovis and mutans streptococci (Mashburn-Warren et al., 2010). The predicted ComS pro-peptides from these species had a similar features, being composed by a high degree of hydrophobic residues, possessing a net positive charge and a double tryptophan (WW) motif in their C-terminal region. Characterization of the ComRS system in *S. mutans* (that interestingly, has both ComDE and ComRS systems) replicated the results seen in *S. thermophilus*, and additionally showed that a synthetic pheromone consisting of the last 8 C-terminal amino acids of the 17 amino-acid long ComS peptide was able to trigger *sigX* expression and transformation in an Opp- and ComR-dependent manner, mimicking the activity of the putative mature pheromone. This active pheromone was named XIP for *sigX*-inducing peptide.

The effect of ComRS signaling in GAS and other pyogenic-group Streptococci, and its effect over transformation have proven to be more cryptic than in non-pyogenic streptococcal counterparts. Even though genome sequencing data has shown that horizontal transfer and genetic exchange between GAS and other streptococcal species is common, the demonstration of natural transformation of GAS in laboratory conditions has proven elusive. Genomic analysis by Mashburn et al. found *comRS* genes in all sequenced GAS genomes, and two allelic variants named M1 and M3 were found. Between these variants, ComR has a highly conserved N-terminal region, indicating similar DNA-sequence binding, but distinct C-terminal regions, suggesting a differential interaction with pheromones (Mashburn-Warren et al., 2012). Indeed, the sequence of XIPs of M1 and M3 alleles also differ, while both contain WW motifs (Table 1). In the absence of exogenous XIP, *sigX* expression is low. Addition of synthetic XIP activates ComR and triggers expression of *sigX* in GAS strains, in an allele-specific manner. Concentrations as low as 0.5 nM XIP are sufficient to trigger *sigX* expression, and microarray analysis revealed that XIP sensing by ComR generated the upregulation of 30 different genes, 21 of these containing com boxes, and thus regulated directly by SigX (Mashburn-Warren et al., 2012). Most of the regulated genes corresponded to predicted competence late genes. Two other regulated genes of interest were *murM2* and *rocA*, involved respectively in processes of antimicrobial resistance and upregulation of the CovR TCS component, a major regulator of GAS genes. All gene functions known to be required for genetic transformation in *S. pneumoniae* and *Bacillus subtilis* were upregulated in response to XIP in GAS, but nonetheless transformation was not seen *in vitro*. Further experiments revealed that competence was blocked at the DNA uptake process, suggesting that unknown post-transcriptional regulation events were inhibiting transformation (Mashburn-Warren et al., 2012). Additional environmental signals may therefore be required to unblock the competence machinery, and the first evidence for this notion has been associated with growth in biofilms. As sessile bacterial aggregates, biofilms contain high densities of bacterial cells in close contact and studies have shown that Streptococci that undergo natural transformation do so in higher efficiencies when growing inside biofilms (Marks et al., 2012; Wei and Håvarstein, 2012). Recently, the first report of effective GAS natural transformation in the laboratory was seen in biofilm cultures (Marks et al., 2014). GAS biofilms grown on a substrate of fixed epithelial cells were shown to internalize and incorporate exogenous DNA at a low frequency, in a ComR-dependent manner. Addition of XIP generated a ten-fold increase in efficiency, but was not required for transformation, suggesting that growth on epithelia provides a signal(s) for Com system activation. The transformation phenotype could also be replicated during an *in vivo* biofilm growth assay, where intranasally colonized mice were also given donor DNA. Again, the addition of the XIP pheromone generated a ten-fold increase in the number of transformed colonies (Marks et al., 2014). These results suggest that GAS are indeed capable of undergoing genetic transformation, but an unidentified regulatory check-point requires an environmental condition that appears to be satisfied through growth on host cells. Also, a number of GAS isolates have reported mutations in the genes that form the DNA uptake complex, indicating the ability to induce the complete competence machinery is naturally lost in some of them, however the conserved maintenance of genes involved in the QS-signaling process suggests that ComR and SigX may have additional beneficial functions in non-competent strains (Woodbury et al., 2006; Mashburn-Warren et al., 2012).

## Sil signaling system

**Figure 3:**
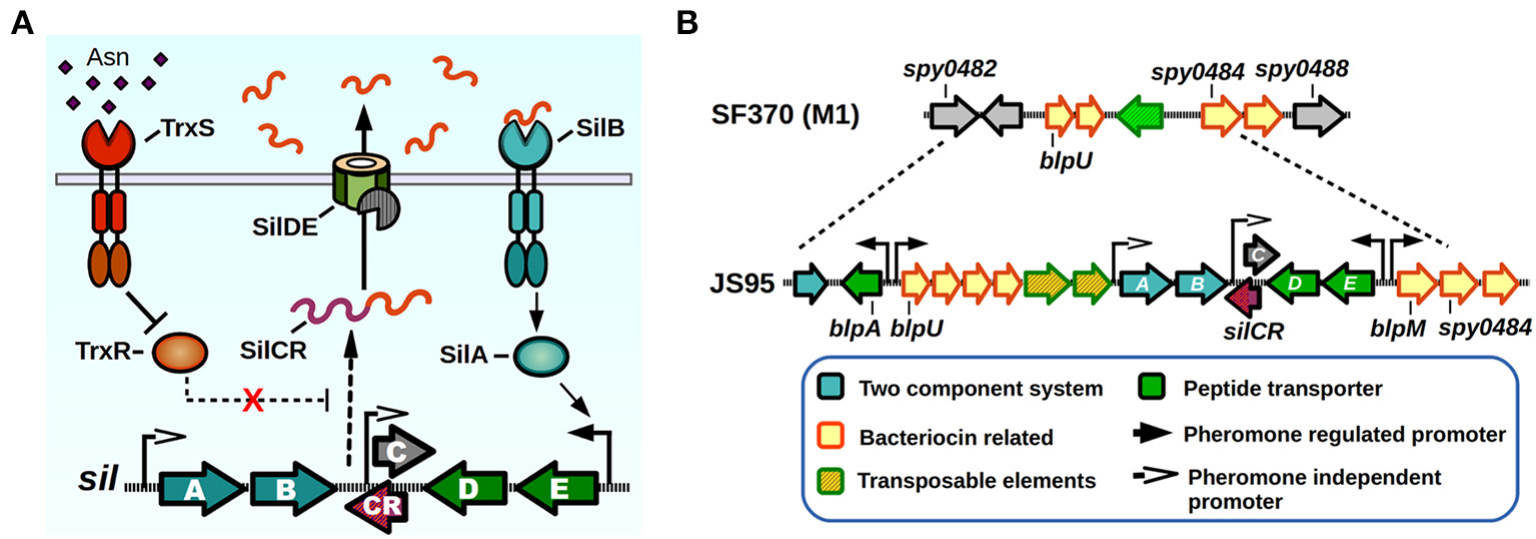
**Sil signaling system. (A)** Model of signaling. SilCR pre-peptide is produced, secreted and processed. Mature SilCR is detected by SilB, activating the response regulator SilA which activates expression from select promoters, including the promoter for the expression of *silED-CR* genes. Expression of the *sil* locus is also in a unknown manner by a second two-component system, TrxSR. Asparagine sensing by TrxS alleviates repression of target genes by TrxR, generating an increase in expression of SilCR dependent promoters. **(B)** *sil* genomic island. The *sil* locus plus neighboring genes are located in a putative genomic island in the strain JS95. Chromosomal location is compared with the SF370 strain that does not possess the *sil* locus.

The Sil system was the first QS network characterized in GAS. The *sil* locus was discovered in a tagged-transposon mutagenesis screen performed on a highly invasive M14 strain (JS95), which was isolated from a case of necrotizing fasciitis, and is capable of generating a lethal invasive infection in a murine model (Hidalgo-Grass et al., 2002). The screen was designed to identify mutant clones that lost their ability to spread invasively from a skin infection site to other target organs in mice. One of the isolated mutants lost its ability to reach the spleen, while not affecting its ability to survive in blood, and had an insertion in a novel locus that was termed streptococcal invasion locus (*sil*). The core Sil system consists of six genes, *silABCDE* and *silCR* encoded in a putative genomic island of 15–17 Kbps in size, comprising a lower GC content than the average of the GAS genome (Hidalgo-Grass et al., 2002; Eran et al., 2007; Belotserkovsky et al., 2009) (Figure 3). The presence of *sil* genes in GAS genomes is not widespread, with only four of the 19 sequenced GAS genomes contain this system (MGAS8232, MGAS10750, Alab49, and HSC5) (Kizy and Neely, 2009; Michael-Gayego et al., 2013). Epidemiological studies have shown that the prevalence of Sil ranges from 12% in non-invasive isolates to 16–25% in invasive isolates, and that the locus is restricted to a few *emm*-types with M4 being the most common *sil*-harboring isolate (Bidet et al., 2007; Billal et al., 2008; Michael-Gayego et al., 2013). The sequenced strains that do not possess Sil have instead remnants of the genomic island, suggesting that DNA recombinations may have been responsible of loss of *sil* genes (Figure 3B) (Belotserkovsky et al., 2009).

The Sil system has sequence homology with the competence (Com) and the bacteriocin-like peptide (Blp) QS systems of *Streptococcus pneumoniae* (De Saizieu et al., 2000; Morrison and Lee, 2000). The *sil* QS locus (Figure 3A) is composed of *silAB*, a putative TCS, *silDE*, a putative ABC transporter, and *silCR*, the pheromone pro-peptide gene. An additional small ORF encoded in the complementary DNA strand and overlapping with *silCR* is *silC*, which encodes a 39 amino acid peptide disrupted by the original transposon hit during the mutant screen (Hidalgo-Grass et al., 2002). The promoter of *silC* has a *com* box, the conserved sequence found in promoter regions of genes involved in competence establishment in other streptococci (Morrison and Lee, 2000). The *silCR* gene encodes a 41 amino acid pro-peptide with a glycine-glycine sequence motif, thought to direct cleavage in a manner consistent with Gly-Gly bacteriocin processing, which allows cleavage into a 17 amino acid active mature pheromone (Table 1). In strains carrying a functional copy of *silCR*, basal pheromone production in standard culture conditions can be quantified by immunoblotting methods and measured in supernatants using fluorescence reporter assays, with a concentration of approximately 24.5 nM being produced by the IB7 GAS isolate (Belotserkovsky et al., 2009). The multiple SilCR-regulated promoters in the *sil* locus are induced to various levels and the amount of synthetic pheromone required to trigger their expression ranges from low to high nanomolar concentrations (Eran et al., 2007; Belotserkovsky et al., 2009). The *silDE* and *silCR* genes are transcribed in a single mRNA whose expression increases in response to synthetic SilCR pheromone. Detection of SilCR is believed to occur through direct interaction of the peptide with the SilB TCS receptor which generates a corresponding phosphotransfer to the SilA and consequent change in DNA expression. The *silAB* promoter on the other hand, shows a lower degree of response to the peptide pheromone, but increases in response to H_2_O_2_, and is upregulated upon inoculation of the host. This suggests, that much like production of other pheromones in GAS, environmental conditions or host factors may be key to triggering their expression (Eran et al., 2007; Belotserkovsky et al., 2009; Kizy and Neely, 2009).

SilA is the predicted TCS response regulator and a member of the AlgR/AgrA/LytR family of transcriptional regulators that bind DNA as dimers via a LytTR-type domain (Nikolskaya and Galperin, 2002). SilA was shown to be necessary together with SilB, the putative sensor histidine kinase, to trigger expression of genes in response to SilCR (Belotserkovsky et al., 2009). The binding sequence for SilA was mapped in two SilCR responding promoters, revealing the presence of two direct-repeats of 9 bp that are separated by an 11 bp spacer, both of which are required for SilA activity. As demonstrated by bioinformatic analysis, most of the genes that possess putative SilA binding sites are present in the genomic island surrounding the *sil* core genes (Figure 3B). These genes increase their expression in a rapid manner after SilCR pheromone addition, and several correspond to predicted bacteriocins and/or bacteriocin maturation components, while the rest of them correspond to transposons or insertion sequences (Belotserkovsky et al., 2009). In *Streptococcus pneumoniae* the Blp bacteriocins were shown to mediate intra- and interspecies competition in cultured conditions, and also provide a competitive advantage during *in vivo* colonization experiments (Dawid et al., 2007). It is unknown whether the Blp homologs found in the *sil* island are able to produce a mature, active bacteriocin, or if they can generate immunity against Blp bacteriocins of *S. pneumoniae*, which have been shown to kill GAS *in vitro* (Lux et al., 2007). Interestingly, addition of SilCR to a culture's growth medium also increases the expression of several genes that lack SilA binding motifs. As shown by microarray experiments, the early response to SilCR addition consists mainly of genes in the *sil* island. However, 3 h after pheromone addition other genes show a moderate but significant response. Some of these genes include *ptsABCD*, a putative mannose/fructose-specific phosphotransferase system, purine synthesis related genes *purEK* and even the *rgg2* transcriptional regulator, suggesting a possible link in the activation process of these two QS systems (Belotserkovsky et al., 2009). Strains that do not possess the core *sil* signaling genes have also been shown to exhibit differential gene expression in response to SilCR pheromone, in some cases with opposite regulatory effects than those seen in *sil* harboring strains (Salim et al., 2008). If any of the additional TCS of GAS could be also sensing SilCR has yet to be determined.

As mentioned earlier, only a fraction of GAS isolates carry the *sil* locus. Interestingly, among the strains that possess Sil, some of them also carry point mutations in components of the system. The pheromone-coding gene, *silCR*, often has a nonsense mutation that changes the ATG start codon for ATA, rendering the strain unable to produce its own peptide, yet is able to respond to exogenous peptide (i.e., strains JS95, HSC5). Other mutations reported are located in the *silD* component of the ABC transporter, where stop codon mutations truncate or divide the gene (strains MGAS8232, MGAS10750) (Michael-Gayego et al., 2013). The strains carrying these *silD* mutations should be able to respond to peptide, but won't be able to secrete pheromones to generate an autoinduction process. Presence of non-sense mutations in signaling pathways appears to be a recurring theme among GAS QS systems, and also can be seen in the Com and the lantibiotic regulatory systems of GAS (Upton et al., 2001; Wescombe and Tagg, 2003; Mashburn-Warren et al., 2012; Wescombe et al., 2012). Selective pressures leading to such lesions remains unclear.

The clearest understanding of behavior regulated by the Sil system, and the reason for its discovery, is its effect on pathogenicity. After the first study which identified *silC* mutants with deficient invasion of the spleen, *silB* and *silC* mutants were also isolated in an independent signature-tagged mutagenesis screen looking for loss of virulence of the strain HSC5 in a zebra fish model of necrotizing fasciitis (Kizy and Neely, 2009). Interestingly, these screens have been performed in strains possessing the *sil* locus, but contain the sequence ATA in place of a functional start codon in *silCR*, impairing pheromone production. Thus, Sil seems to provide a pathogenic advantage *in vivo* in the *absence* of the pheromone, and further evidence suggests SilCR pheromone actually represses pathogenicity. Normally, GAS exhibits the ability to degrade the chemokine IL-8 of human origin, and MIP2 and KC of murine origin, by the activity of the ScpC/SpyCEP serine peptidase. Chemokine degradation impairs the process of immune recruitment, resulting in the absence of infiltrating neutrophils in infected mice tissue, thus inhibiting bacterial clearance from infected sites. Preincubation of the strain JS95 with the SilCR pheromone inhibits the chemokine-degrading activity by downregulating the expression of ScpC in a SilA-dependent SilC-independent manner, and preincubation of bacteria with SilCR reduces skin lesion area and inhibits killing by systemic infection (Hidalgo-Grass et al., 2004, 2006; Eran et al., 2007). For this reason it has been proposed that the Sil system may play a role in controlling invasive disease, and that strains possessing Sil, but have mutations impairing SilCR expression, may exhibit increased virulence phenotypes. The mechanism of ScpC downregulation has not been elucidated thus far, and its promoter lacks any predicted SilA binding sites. Even though its deletion affects pathogenicity, the exact function and mechanism of SilC remains cryptic. Originally, it was shown that the expression of SilC *in trans* from a plasmid was able to repress SilCR activated promoters (Hidalgo-Grass et al., 2002; Eran et al., 2007), but so far its ability to repress other genes that may affect GAS infection and lifestyle in the host has not been demonstrated. The therapeutic effect of SilCR nonetheless seems to be variable, and may depend on the presence of *sil* genes, emm type and isolate used (Salim et al., 2008), and repression of *scpC* expression by additional regulators like CovRS (Sumby et al., 2008). Recently, it was shown that signaling by Sil is activated *in vivo* during early time-points post infection in a mouse model, and that this activation phenotype and its kinetics can be replicated in culture during infection of cell-culture lines (Baruch et al., 2014). It was shown that activation of the *sil* locus was dependent on the sensing of the amino acid asparagine by the TrxRS TCS of GAS, subsequent to secretion of streptolysin toxins that induce asparagine release by mammalian cells.

Finally, isolates of *Streptococcus dysgalactiae subsp. equisimilis*, known as Group G streptococcus (GGS), also possess the *sil* locus. Prevalence of Sil in invasive isolates of GGS is higher than in GAS isolates, and all analyzed strains had a functional copy of *silCR*, although some of them also carried truncations in the *silD* gene (Belotserkovsky et al., 2009; Michael-Gayego et al., 2013). Both GAS and GGS generated a transcriptional response when incubated with SilCR pheromone produced by one another, supporting the hypothesis that interspecies signaling between these two Streptococci is possible.

## Lantibiotic regulatory systems

**Figure 4:**
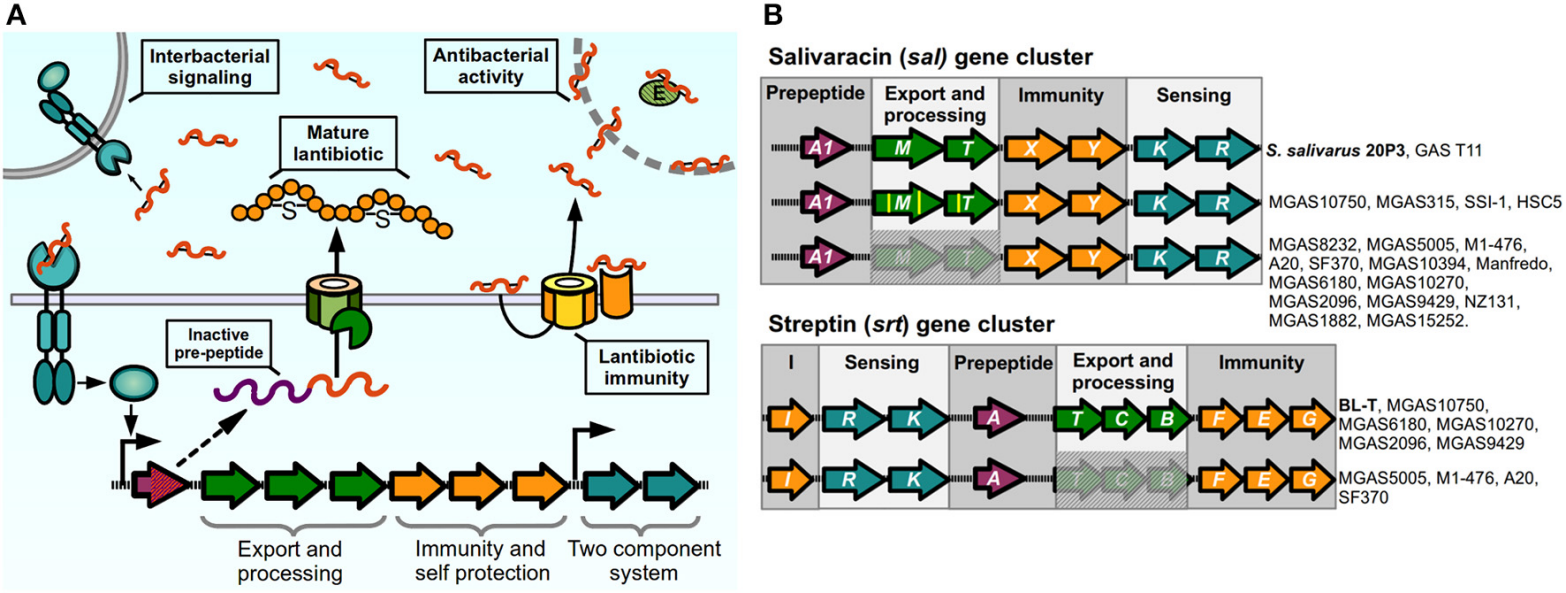
**Lantibiotic regulatory systems. (A)** Model of production and sensing. Lantibiotic pre-peptide is synthesized, exported, cleaved, and modified through thiosulfur bridge formation and aminoacid dehydration. The mature lantibiotic can exert its antibacterial effect on sensitive bacteria either by targeting the cytoplasmic membrane or inhibiting activity of target enzymes. Lantibiotic-producing bacteria express membrane immunity proteins to bind their cognate lantibiotic, or to re-export via an ABC transporter. Two-component systems sense the lantibiotic and trigger activation of the lantibiotic gene cluster in producing and neighboring bacteria. **(B)** Lantibiotic gene clusters present in *Streptococcus pyogenes*. Complete or partial components of lantibiotic genes in sequenced strains of GAS compared with reference strains (in bold) for *sal* cluster (*S. salivarius* 20P3, accession AY005472), and *srt* cluster (*S. pyogenes* BL-T, accession AB030831).

During the process of colonization and establishment of their niche, bacteria face constant competition for nutrients from other individuals. For this reason, several bacteria have devised systems to give them a competitive advantage against other bacterial organisms. One such way is the production of bacteriocins, ribosomally synthesized antimicrobial peptides (AMP) that can target bacteria in the same species or across genera, with producing bacteria expressing specific immunity proteins to protect themselves from their cognate peptide. These antimicrobial molecules can act through a variety of mechanisms including membrane pore formation, cell wall synthesis inhibition, and target enzyme inhibition (Quadri, 2002; Cotter et al., 2005). Class I bacteriocins, termed lantibiotics, are post-translationally modified peptides that are produced by Gram-positive bacteria, including *Bacillus, Staphylococcus, Lactococcus* and *Streptococcus* species (Chatterjee et al., 2005). These molecules are synthesized as inactive pro-peptides and then modified through amino acid dehydration and/or thioether bridge formation to generate unusual amino acid residues, and are cleaved during the secretion process to generate a mature active lantibiotic (Kleerebezem, 2004; Chatterjee et al., 2005). The lantibiotic acts not only as an AMP to neighboring bacteria, but also has pheromone properties, as lantibiotic-producing bacteria can detect their own bacteriocin through a TCS. For these reasons, lantibiotic production in several bacteria exhibits a cell-density dependent pattern and is regulated by quorum-sensing like circuits (Kleerebezem, 2004). The machinery required for lantibiotic production is usually encoded in gene clusters of conserved architecture formed by two or more operonic units in which genes are grouped by their function. A model of a prototypical lantibiotic producing system and its mechanism is shown in Figure 4A: The inactive product of the pre-peptide gene is post-translationally modified, cleaved, and exported to give rise to the active lantibiotic, which can exert is antimicrobial activity toward sensitive individuals. The mature lantibiotic is also sensed by the TCS, which signals to generate an autoinduction process and increase the production of lantibiotic as well as activates the production of lantibiotic in its siblings. In some cases lantibiotic biosynthetic operons are shared amongst different species, meaning that a bacteriocin can act both as an inter and intraspecies signaling molecule. To remain resistant to the activity of their lantibiotic, bacteria can express membrane bound immunity peptides that bind the bacteriocin and/or ABC transporters that prevent the accumulation of lantibiotics in the bacterial surface (Gebhard, 2012).

Certain GAS isolates have been reported to produce different lantibiotic molecules that can inhibit growth in other streptococcal species, or in sensitive GAS isolates (Tagg et al., 1973; Simpson and Tagg, 1983; Tagg and Skjold, 1984; Hynes and Tagg, 1985; Karaya et al., 2001; Upton et al., 2001). The ability to produce mature bacteriocins is not widespread amongst all GAS isolates, and in most cases conserved mutations have occurred in biosynthetic loci. This has led authors to hypothesize that some of these circuits may have lost their lantibiotic and QS ability and instead have adapted them to perform other functions (Phelps and Neely, 2007; Namprachan-Frantz et al., 2014). One lantibiotic locus present in GAS is orthologous to Salivaracin A (SalA) synthesis gene clusters. First identified and purified form cultures of *Streptococcus salivarius* strain 20P3, salivaracin A is the result of the processed product of the *salA* gene, a 51-amino acid propeptide with a Gly-Gly motif, which is modified and cleaved to generate a mature 22-amino acid lantibiotic and autoinducer pheromone (Ross et al., 1993; Upton et al., 2001). Different *sal* alleles have been described where variations in the sequence of the *salA* gene give rise to five additional variants of SalA termed SalA1 to SalA5 (Wescombe et al., 2006). The *S. salivarius salAMTXYKR* locus contains all the genes predicted to be required for the processing (*salM*, previously mistakenly annotated as *salBC*), export (*salT*), immunity (*salXY*), and sensing of the peptide (*salKR*) by the SalKR TCS (Ross et al., 1993; Wescombe et al., 2006). Salivaracin A from *S. salivarius* was shown to inhibit growth of a wide variety of GAS strains in culture. Surprisingly, the majority of analyzed GAS isolates were also shown to possess a *salA1* gene in their genome, while only a couple of M4 isolates were able to produce the active lantibiotic (Simpson et al., 1995; Upton et al., 2001; Wescombe et al., 2006). Analysis of genomes of these isolates revealed that most of them carried deletions in the *sal* cluster affecting the genes required for maturation and secretion of SalA (Wescombe et al., 2006), while mostly maintaining intact sequences of the downstream genes required for immunity and sensing, a phenomenon that seems to be widespread as shown also in several sequenced strains (Figure 4B). Spent cultures of a the GAS T11 strain, that is able to produce active SalA1, induces expression of *salA* mRNA in *S. salivarius* 20P3, as well as inducing low levels of expression of *salA1* mRNA in the M1 SF370 strain, which has an incomplete *sal* locus, meaning that this strain is still able to sense and generate a transcriptional response to the lantibiotic peptide. Nonetheless, SF370 remains sensitive to the bacteriostatic effects of SalA, although it carries the genes for its putative immunity, suggesting that the ability to generate an active lantibiotic and the process of self-signaling and autoinduction are required for efficient establishment of immunity. Reinforcing this idea, mutating the SalA maturation genes in the immune strain T11 renders it sensitive to SalA. Some GAS strains possess all the genes of the *sal* cluster (MGAS10750, MGAS315, HSC5, SSI-1), although point mutations in maturation and transport genes may still render these strains unable to produce the active lantibiotic and are sensitive to its activity (Wescombe et al., 2006; Phelps and Neely, 2007; Namprachan-Frantz et al., 2014). Evidence has suggested however that the *sal* locus may still play a role during GAS infection in the host. Analysis of GAS transposon mutants in a zebrafish model of infection detected two attenuated mutants of the HSC5 strain with insertions in *salY* and *salK* genes. Further inspection showed that the *salY* mutant exhibited decreased bacterial loads after zebrafish infection when compared with is WT counterpart, and the *salY* mutant was also attenuated for survival inside macrophages *in vitro*. The *in vivo* survival defect could be rescued if zebrafish were depleted of macrophages prior to infection (Phelps and Neely, 2007). SalY forms part of the putative ABC transporter involved in immunity to the SalA lantibiotic. When compared with the *salY* gene from *S. salivarius*, all GAS strains possess conserved nucleotide changes that generate non-similar changes in several amino acids of the SalY transporter. Therefore, it has been hypothesized that GAS SalY may have changed and adapted to bind and/or export other substrates. It has also been shown that *salA* expression is upregulated during murine muscle tissue infection, and recently it also was shown that human serum increases the expression of the promoter upstream of the salRK TCS genes, providing a possible link between host factors and activation of the *sal* locus in GAS (Loughman and Caparon, 2006a; Namprachan-Frantz et al., 2014). In addition, data have suggested that mutations in the salRK TCS can affect other genes outside the *sal* cluster of genes in GAS and other streptococci (Li et al., 2008; Le Breton et al., 2013). For these reasons, it was hypothesized that while the antimicrobial effect of SalA1 was lost in GAS, it may still be involved in signaling processes important for host colonization, and that this system may have evolved to perform a different function.

Another lantibiotic isolated from GAS cultures is Streptin (Karaya et al., 2001; Wescombe and Tagg, 2003). Similar to salivaracin A and other lantibiotics, all the required genes are present in one cluster, formed by the *srt*IRKATCBFEG genes predicted to be involved in streptin binding and immunity (*srtI*), detection and regulation by TCS (*srtRK*), pre-peptide (*srtA*), transport (*srtT*), modification (*srtCB*), and additional immunity through an ABC-transporter (*srtFEG*). The *srtA* gene encodes for the Streptin precursor peptide, a 46 amino acid peptide that is modified and cleaved to generate three variants of Streptin (Table 1) (Wescombe and Tagg, 2003). Levels of *srtA* mRNA are undetected at early culture time points and increases slightly at later stages of culture, but can be induced earlier if a streptin extract is added to the culture, reflecting the autoinducing ability of the lantibiotic. Similarly, potent streptin production is only obtained in two-phase cultures, where a lawn of solid media-grown bacteria is transferred to liquid culture. Interestingly, presence of SpeB has been shown to be required to produce active Streptin and to induce effective immunity in the producer strains, suggesting that this protease may be involved in the processing of the mature lantibiotic (Hynes and Tagg, 1986). Though the *srtA* gene is found in several isolates, only a few of these are able to produce the biologically active lantibiotic, as is seen with SalA. In some cases lack of production is apparently due to the lack of genes required for transport and modification, or in other cases where the *srt* locus appears intact and autoinduction does not occur due to probable lack of promoter activity, suggesting that additional elements are involved in controlling expression of the lantibiotic genes (Wescombe and Tagg, 2003). One group has shown that samples of GAS present in throat infection patients exhibit a high expression of the *srtEGI* immunity genes (Livezey et al., 2011), suggesting a requirement of host factors for *srt* gene expression and a possible role for streptin immunity-related ABC-transporters in infection of the host and establishment of GAS in its niche.

Finally, Streptococcins are another type of lantibiotic shown to be produced by GAS. There have been two types described to date, class I type streptococcins (chromosomally encoded Streptococcin A-FF22 and Streptococcin A-M49, named so for their strains of isolation) and class III type streptococcins (plasmid encoded Streptococcin A-M57) (Tagg et al., 1973; Simpson and Tagg, 1983; Tagg and Skjold, 1984; Jack and Tagg, 1992; Hynes et al., 1994; Jack et al., 1994b; Heng et al., 2004; Tagg, 2009). The most studied of these is Streptococcin A-FF22 (SA-FF22) a cationic bactericidal peptide which causes membrane potential disruption in target cells (Jack et al., 1994a). Similar to the *sal* and *srt* loci, the gene cluster involved in SA-FF22 is comprised of genes involved in production of the pre-peptides (*scnA* and *scnA*′), regulation (*scnRK*), modification and transport (*scnMT*), and immunity to the lantibiotic (*scnFEG*) (McLaughlin et al., 1999). The FF22 strain is able to produce an active Streptococcin that is able to inhibit growth in sensitive strains, while generating autoinduction in both GAS and *S. salivarius* and *S. dysgalactiae* strains that carry the complete *scn* gene cluster. The *scnA* pre-peptide gene was found only in a small percentile of analyzed strains, and all of the sequenced strains to date completely lack the locus, having only a flanking transposase A gene (Wescombe et al., 2012), and studies testing the streptococcin involvement in the GAS lifestyle have not been reported.

## Interspecies communication: LuxS and AI-2

**Figure 5:**
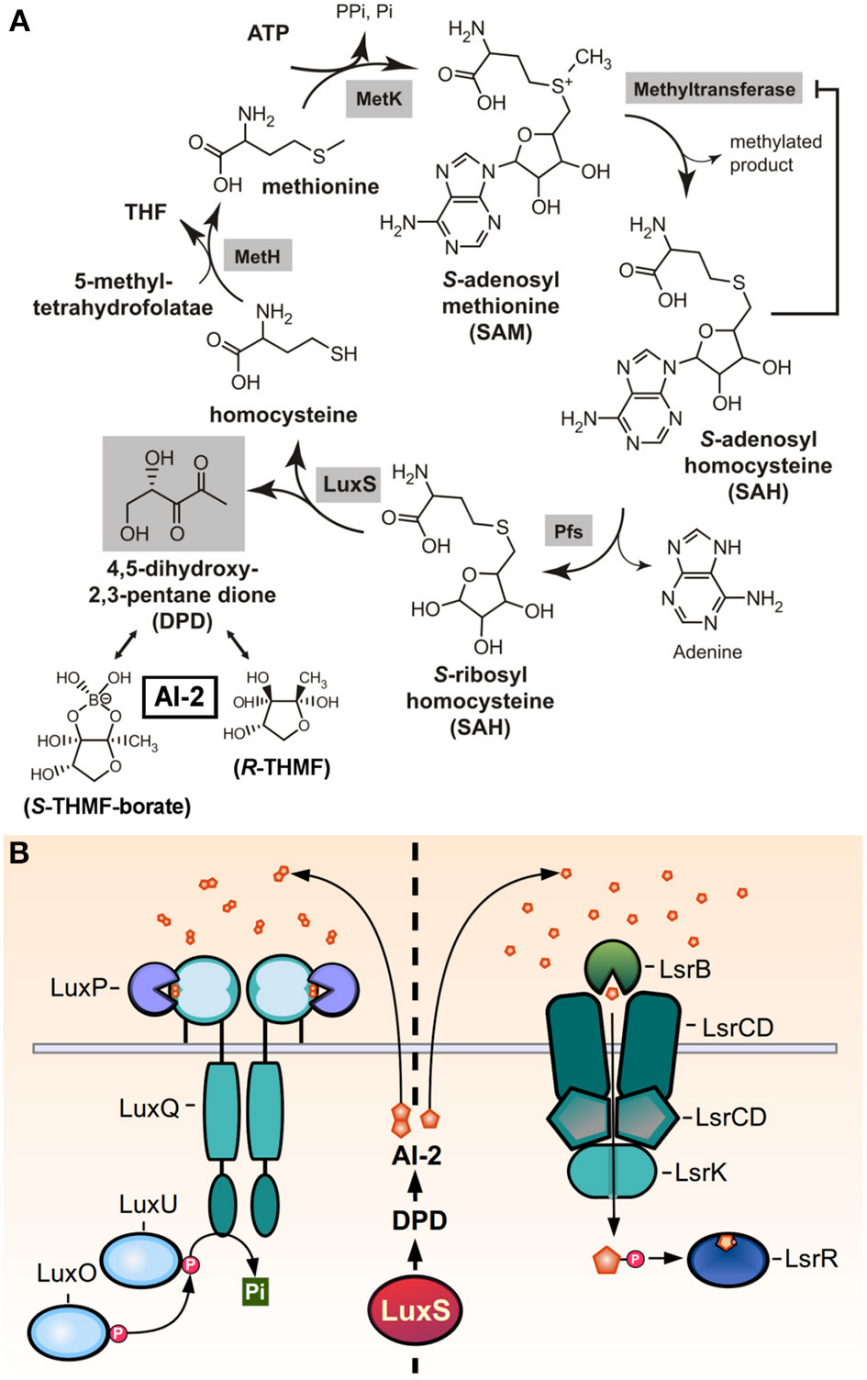
**AI-2 production and sensing. (A)** Reactive methyl cycle and production of DPD. SAM-dependent methyltransferases convert SAM to SAH, accumulation of which confers product-feedback inhibition on methyltransferase reactions. SAH is detoxified to SRH by Pfs. SRH is converted to homocysteine and DPD by LuxS. Homocysteine can be recycled to SAM via MetH, which generates methionine, and MetK. DPD spontaneously cyclizes and undergoes ketone hydration to form *R*- and *S*-THMF. The latter is also found in its borated-form, *S*-THMF-borate (reproduced and modified with permission from Federle, 2009) **(B)** The two families of known AI-2 receptors, LuxPQ, and LsrB, which sense extracelullar AI-2 or import AI-2 to bind LsrR, respectively. LuxPQ binds the *S*-THMF-borate form of AI-2, while LsrB binds the *R*-THMF form.

The autoinducer-2 molecule (AI-2) was initially discovered in the Gram-negative bacterium *Vibrio harveyi*, in which it forms part of the QS-dependent regulation of luciferase production. It was later found that the LuxS enzyme was required for AI-2 production, and since the *luxS* gene is present in the genomes of a wide variety of Gram-negative and Gram-positive bacteria, has led to the proposal of AI-2 as a universal signal for interspecies cell-to-cell communication (Miller and Bassler, 2001; Xavier and Bassler, 2003). Differing from other Gram-positive QS signals, AI-2 signaling is not peptide-based. Also, unlike the biosynthetic processes of the other QS molecules of GAS, the AI-2 synthesis pathway is tightly coupled with a metabolic cycle termed activated methyl cycle (AMC), which is involved in the utilization of *S*-adenosylmethionine (SAM) and the degradation of its toxic derivatives. SAM is an essential donor of methyl groups for processes of DNA, RNA, fatty acid, and protein synthesis. Its utilization generates the toxic intermediate *S*-adenosylhomocysteine (SAH), which is then hydrolized by the Pfs nucleosidase to produce S-rybosylhomocysteine (SRH) and adenine. LuxS catalyzes the cleavage of SRH to homocysteine and 4,5-dihydroxy 2,3-pentanedione (DPD). DPD then spontaneously cyclizes to form pro-AI-2 molecules capable of reacting with borate to generate the signal detected by Vibrios (Figure 5A) (Schauder et al., 2001).

Two different families of receptors have been reported to sense AI-2 molecules in different bacteria (Figure 5B). The LuxPQ family, only found in *Vibrio* spp., consisting of the LuxP periplasmic protein that binds AI-2 and in consequence modulates the activity LuxQ, a transmembrane two-component histidine kinase. Binding of AI-2 switches the activity of LuxQ from kinase to phosphatase, affecting the activity of downstream DNA-binding response regulators. The other family of receptors is the LsrB family, found in *E. coli*, *Salmonella* and members of the *Rhizobiaceae* and *Bacillaceae* families (Pereira et al., 2009). LsrB binds a non-borated derivative of the AI-2 molecule, but unlike the LuxPQ signal transduction system, LsrB delivers AI-2 to the Lsr ABC transporter, which imports the molecule into the cytoplasm. The transport of AI-2 it's coupled with its phosphorylation, which both serves to its sequestration inside the cell and to enhance AI-2 binding to the LsrR transcriptional repressor protein, deactivating its repressive function (Miller et al., 2004; Xavier and Bassler, 2005).

LuxS and AI-2 have been shown to influence expression of virulence factors, motility, and biofilm formation in several bacteria, including Streptococcal species (Reviewed by Pereira et al., 2013). In GAS, LuxS is required for AI-2 synthesis, and *luxS* deletion resulted in increased expression of the haemolysin streptolysin S and Emm protein, decreased production of hyaluronic acid capsule and diminished activity of the SpeB protease due to abnormal processing (Lyon et al., 2001; Marouni and Sela, 2003). These changes resulted in increased invasiveness toward HEp-2 epithelial cells. Additionally, Siller et al. showed that the deletion of the *luxS* gene resulted in increased tolerance to acidic conditions, affected the expression of *fasX* (an sRNA involved in regulation of virulence factor expression), (Kreikemeyer et al., 2001) and upregulated expression of *sibA*, a gene encoding a secreted immunoglobulin binding protein (Siller et al., 2008).

One of the central debates in the topic of LuxS function is how to differentiate its QS effects from its metabolic role. Inactivation of *luxS* not only inhibits AI-2 formation, but it also could result in gene expression changes generated by defective recycling of homocysteine or accumulation of intermediates of SAM metabolism. Recently, Redanz et al. have shown that in *Streptoccus sanguinis* close to 96% of the genes that exhibit a change in expression in the Δ*luxS* mutant are due to accumulation of homocysteine in the cell (Redanz et al., 2012). Additionally, genomic analysis has revealed that several species possessing the *luxS* gene lack any homologs to the AI-2 receptors LuxPQ or Lsr, including the Streptococci (Rezzonico and Duffy, 2008). Thus, it has been proposed that to clearly establish the role and the effects of the AI-2 system, the Δ*luxS* mutant phenotypes should be complemented by the addition of synthetic DPD to generate AI-2 in the culture medium (Pereira et al., 2013). No group has yet demonstrated directly the effects of exogenous AI-2 on GAS gene expression, so the nature of the LuxS-promoted phenotypes is unknown. Even though Siller et al. have suggested that *luxS* has a limited role in the AMC cycle in GAS (Siller et al., 2008), the overall effect of this deletion over the bacterial metabolism is unknown. Nonetheless, AI-2 has been shown to directly influence biofilm formation in other oral streptococci like *S. intermedius* and *S. gordonii* (Ahmed et al., 2008; Cuadra-Saenz et al., 2012), meaning that these bacteria may have an as yet uncharacterized AI-2 sensing machinery, and that even if GAS does not directly respond to its own AI-2, could influence the behaviors of related species in its surroundings.

## Concluding remarks and perspectives

The *Streptococcus pyogenes* pan-genome harbors several varieties of QS circuits, and GAS has the ability to produce an assortment of dedicated signaling molecules that affect expression of several target genes. While there is increasing information regarding the mechanics of these QS networks, our current understanding of these systems is still in its infancy, and despite data regarding regulated genes and observed phenotypes resulting of QS signaling, we do not fully comprehend yet when and where these communication systems are triggered *in vivo*, and how GAS benefits from these processes of population-wide coordination of gene expression. Apart from the Sil system, the direct contribution of QS to GAS pathogenesis is unclear. However, its putative involvement in the control of DNA exchange (ComR) and in the sensing and production of antimicrobial substances (SalA and Srt) suggest that QS may play roles involved in the less understood process of GAS interaction with other bacteria in its milieu. Recently, Marks et al. have shown that GAS growing inside biofilms exhibit lower expression of genes involved in localized and invasive-disease, and bacteria originating from biofilm cultures had reduced virulence in a septic infection model, while showing an increased ability to asymptomatically colonize nasal associated lymphoid tissue of mice, when compared with planktonic cultured bacteria (Marks et al., 2014). There is evidence that both Rgg2/3 and Sil QS systems can affect biofilm formation (Lembke et al., 2006; Chang et al., 2011; Cook et al., 2013), suggesting a possible link between QS and the asymptomatic, commensal lifestyle of GAS inside the human host. Future efforts in these areas may be able to reveal the role of QS signaling in GAS population behavior.

With the increasing emergence of antibiotic resistance amongst infectious bacteria, there is a dire need for alternative therapeutic strategies to control pathogens. One of those strategies is the control of virulence gene expression, mainly through the manipulation of QS in a process of quorum-sensing inhibition (also referred to as “quorum quenching,” reviewed by LaSarre and Federle, 2013), with successful results of QS-inhibition during *in vivo* infection models with pathogens *Vibrio cholerae* and *Staphylococcus aureus* (Mayville et al., 1999; Wright et al., 2005; Duan and March, 2010). We think the multiple QS systems of GAS offer interesting molecular targets to block or interfere in order to modulate the behavior of this pathogen as a way of future treatment.

### Conflict of interest statement

The authors declare that the research was conducted in the absence of any commercial or financial relationships that could be construed as a potential conflict of interest.
